# The experiences and barriers in addressing type 2 diabetes mellitus-associated erectile dysfunction: a mixed method systematic review

**DOI:** 10.1186/s13643-023-02303-4

**Published:** 2023-08-10

**Authors:** Setho Hadisuyatmana, Gulzar Malik, Ferry Efendi, Sonia Reisenhofer, James Boyd

**Affiliations:** 1 The School of Psychology & Public Health, La Trobe University, Kingsbury Drive, Bundoora, Victoria 3086 Australia; 2 The Faculty of Nursing, Universitas Airlangga Indonesia, Kampus C Jln Mulyorejo 60115, Surabaya, East Java Indonesia; 3 The School of Nursing and Midwifery, La Trobe University, Kingsbury Drive, Bundoora, Victoria 3086 Australia; 4 Bairnsdale Regional Health Service, 122 Day Street, Bairnsdale, Victoria 3875 Australia

**Keywords:** Type 2 diabetes mellitus, Erectile dysfunction, Systematic review, Barriers, Experience, Screening, Discussion

## Abstract

**Introduction:**

Experiences and determinants connected with type 2 diabetes mellitus-associated erectile dysfunction (T2DMED) in health appointments are not well understood and infrequently reported. This systematic review was undertaken to synthesise evidence of the experiences, facilitators, and barriers around screening ED in men with T2DM during health service consultations.

**Methods:**

The review report was based on the guidelines provided by the Joanna Briggs Institute for conducting mixed-method systematic reviews. Eight electronic databases were searched, including Web of Science, Embase via Ovid, Cumulative Index to Nursing and Allied Health Literature (CINAHL) via EBSCO, ProQuest, PubMed, PsychInfo via Ovid, MEDLINE via Ovid, Portal Garuda. Additionally, the review manually looked through the reference lists of the studies we included. *Erectile dysfunction, type 2 diabetes mellitus*, *screening* and *barriers* were initially used as keywords in the search strategy. All identified primary studies written in English and Bahasa Indonesia, and published between 2001 and 2022 were meticulously screened following an agreed set of inclusion criteria.

**Findings:**

Out of 3468 papers screened, only six were chosen for the review. These included three cross-sectional studies, two qualitative studies, and one mixed-method study. The study quality of the included studies was assessed using the Joanna Briggs Institute (JBI) critical appraisal checklist. Based on the checklist criteria, the studies ranged between 5/10 to 9/10 in terms of quality. After synthesizing the findings, four main categories were identified including the willingness to discuss T2DMED, the barriers experienced and perceived, the limited understanding of T2DMED, and the support expected by men with T2DM.

**Discussion:**

Many men kept quiet about their struggles with T2DMED, hoping to bring it up as a topic of discussion during healthcare consultations. Barriers such as embarrassment, a sense of helplessness and reluctance to seek help, financial constraints, and dismissive healthcare professionals hindered them from addressing this issue. Both the participating men and healthcare professionals lacked a comprehensive understanding of T2DMED.

**Recommendations:**

It is important to provide education tailored to men's specific needs and improve awareness about T2DM-associated ED. Creating a more T2DMED-friendly environment could be a potential solution to increase early screening and management. Future research should investigate potential barriers that prevent HCPs from identifying and addressing T2MED since their absence in the identified studies highlights this need.

**Systematic review registration:**

CRD42021292454.

**Supplementary Information:**

The online version contains supplementary material available at 10.1186/s13643-023-02303-4.

## Introduction

Erectile dysfunction (ED) is a recognised complication of type 2 diabetes mellitus (T2DM) in men [1]. Prolonged interruptions to penile blood flow and progressive neuropathies associated with disease duration, ageing, and psychological distress have been identified as causes of ED [2–5]. T2DM-associated ED (T2DMED) is a contributing factor to a decline in quality of life [6, 7] and an increase in psychological distress and frustration [8]. The condition is often associated with a lack of physical activity and unhealthy dietary habits. In addition, T2DMED is a marker for further medical complications, including increased risk of stroke and cardiovascular diseases (CVD) [9, 10].

Multiple studies have identified that CVD can occur within two to three years following ED diagnosis [11–13]. As a result, early detection of ED and initiation of intensive T2DM management are significant factors in mitigating the potential health threats of CVD [11]. The use of statins and phosphodiesterase-5 inhibitors (PDE5I) in addition to T2DM medication and adoption of healthier lifestyle (including diet change, smoking cessation and physical exercise) [14] are promoted as essential in T2DM management [12]. However, evidence of initiatives to support early detection of T2DMED is limited and currently underreported.

Although multiple T2DM guidelines recommend that men with T2DM should be screened for ED regularly [6, 15, 16] studies have reported challenges associated with discussing ED at healthcare consultations [17]. Based on the available evidence, it seems that there is a lack of willingness and recognition of the importance of discussions about ED between T2DM and healthcare professionals. Additionally, health professionals may not always realize the significance of the issue at hand. This is important because affected men expect to have these discussions initiated by their healthcare professionals [8, 18]. Unfortunately, there is limited understanding and research on these topics. As a result, we conducted this review to gather evidence and synthesize information regarding the experiences and barriers related to discussing T2DMED screening.

In addition, the healthcare professionals as a foundation of the health system does not always recognize the significance of the problem [19]. Nonetheless, thorough investigations on these topics were scattered and less synthesized. Therefore, this review was undertaken to synthesize evidence around the experiences and barriers of screening and discussing T2DMED.

## Methods

### Aims

This systematic review aimed to answer the question: “What are the experiences, facilitators and barriers of screening ED in men with T2DM?” Published studies that investigated the topic using the perspectives of both the healthcare professionals (HCP) and the men were considered for inclusion. The objective was to generate an understanding of the factors that impact ED screening discussions during health appointments, as well as the experiences reported by healthcare professionals (HCP) and men diagnosed with T2DM. The review is driven by a gap in the care provided to T2DMED that has a negative impact on the men’s general and psychological health leading to a poor quality of life [20–22].

### Design

Systematic reviews allow an analysis based on a collection of individual studies on a specific topic [23]. This systematic review was designed and conducted following the Mixed Method Systematic Review (MMSR) informed by the Joanna Briggs Institute [24]. The selection of MMSR was based on the scarcity of articles and was selected to include more potential studies for data extraction and synthesis. The review employed a convergent and sequential [24] approach that included quantitative and qualitative studies to answer the review question [24]. The systematic review reports findings using the 2020’s PRISMA reporting guideline (Supplementary file 1) [25]. The protocol for this systematic review has been registered and accessible in the International Prospective Register of Systematic Reviews (PROSPERO) numbered CRD42021292454 (https://www.crd.york.ac.uk/prospero/).

### Preliminary search

Ten systematic review databases, including PROSPERO, Turning Research Into Practice (TRIP), National Institute for Health and Care Excellence (NICE), Joanna Briggs Institute (JBI), Cochrane Database of Systematic Review (CDSR), Embase via Ovid (Embase), Cumulative Index to Nursing and Allied Health Literature (CINAHL) via EBSCO, MEDLINE via Ovid, PsychINFO via Ovid, and PubMed were explored to ensure that no protocol and review reports had been recently published (or were on-going) on topics related to ED in T2DM population.

### Inclusion criteria

This review included published articles based on the following eligibility criteria:

#### Population(s)

This review adopted the definition of HCPs according to the international standard classification of occupations by the International Labour Organization [26], that includes: medical doctors (generalists, specialist practitioners, public health doctors), nursing professionals (including clinical practitioners, primary health, public health and community health nurses) and pharmacists, midwifery professionals, and dentists [26]. The population in this review is limited to (1) medical doctors, nursing professionals and pharmacist (2)who worked in in-patient and/or out-patient clinics; (3) either in public health services, private services or referral facilities; and (4) men as healthcare and service users.

#### Phenomenon of interests

This review included studies that investigated experiences and challenges in screening or discussing T2DMED.

#### Context(s)

This review included studies that were conducted in all healthcare service settings, including but not limited to hospitals, private practices, and primary care health services.

#### Types of study

This systematic review only considered primary studies, including quantitative, qualitative, and mixed methods, published from 2001 and written in English or Bahasa Indonesia. Mixed method studies were included if the data from quantitative or qualitative components could be extracted separately for analysis. This review did not include discussion papers, editorials, books, secondary studies (such as reviews and meta-analysis), thesis, and grey literature (Table 1).

**Table 1:** Inclusion criteria applied in the present systematic review

*Parameter*	Inclusion criteria	Exclusion criteria
*Population*	HCP including general practitioners, endocrinologists, community health nurses, public health nurses, pharmacists, nutritionists Men diagnosed with T2DM	Dentists, midwives, complementary and alternative medicine practitioners
*Phenomenon of interests*	Screening or assessment and care of men with T2DM related to ED	Men with ED in the general population
*Settings*	Studies that were conducted in health care services, including but not limited to hospitals, private practices, and public health centres	Traditional/complementary and alternative medicines
*Study design*	Qualitative studies using descriptive approaches, phenomenology, case study and grounded theory; quantitative studies including non-experimental designs; and mixed-method studies	Conference abstracts/proceedings, reviews, symposium reports, case reports, comments, editorials, letters to the editor, reviews, books, and grey literature (i.e., government reports, single case reports, strategic documents, expert opinions)

### Search strategy

The search for potential articles was initiated using Scopus by Elsevier, with the initial keywords of “erectile dysfunction”, “type-2 diabetes mellitus”, “screening” and “barriers”. Keywords identified in the titles and abstracts of the relevant articles were then expanded using Medical Subject Headings (MeSH) browser by the National Institute of Health (https://meshb.nlm.nih.gov/). All identified key terms were expanded and the Boolean operators of “AND” and “OR” were used when appropriate (Table 2).

**Table 2:** Initial search terms and databases

1	(Erectile dysfunction or erectile disfunction or impotence or impotent or male impotence or male impotent or male sexual impotence or male sexual impotent or male sexual dysfunction or male sexual disfunction).mp. [mp = title, abstract, heading word, drug trade name, original title, device manufacturer, drug manufacturer, device trade name, keyword heading word, floating subheading word, candidate term word]
2	(diabetes mellitus or type-2 diabetes mellitus or diabetes mellitus type-2 or type-2 diabetes or DM or hyperglycaemia or adult onset diabetes).mp. [mp = title, abstract, heading word, drug trade name, original title, device manufacturer, drug manufacturer, device trade name, keyword heading word, floating subheading word, candidate term word]
3	(screen or screening or discuss or discussion or diagnose or diagnosis or management or manage).mp. [mp = title, abstract, heading word, drug trade name, original title, device manufacturer, drug manufacturer, device trade name, keyword heading word, floating subheading word, candidate term word]
4	(experienc* or barrier or barriers or challeng* or facilitat* or determinan or determinants or treatment or intervention).mp. [mp = title, abstract, heading word, drug trade name, original title, device manufacturer, drug manufacturer, device trade name, keyword heading word, floating subheading word, candidate term word]
5	#1 AND #2
6	#3 OR #4
7	#5 AND #6

* was used as a 'wildcard' in database search to extend potential findings by allowing the journal databases to define the end of the associated word. For instance: the keyword facilitat* will be automatically extended in finding potential articles with keywords facilitate, facilitating, facilitation, facilitations, etc.

### Information sources

Eight databases including Web of Science, Embase via Ovid, Cumulative Index to Nursing and Allied Health Literature (CINAHL) via EBSCO, ProQuest, PubMed, PsychInfo via Ovid, MEDLINE via Ovid, and Portal Garuda, were explored to identify eligible articles. The selection of the databases was designed to encompass the collection of eligible health-related studies. Manual search was conducted against the reference lists from identified articles and assessed for additional inclusion.

### Assessment of methodological quality

All included studies were assessed for methodological quality using JBI’s critical appraisal checklists [24] (Supplementary file 2) [24]. Qualitative articles were appraised using the JBI critical appraisal checklist for qualitative research [27]. Quantitative studies were assessed using the appraisal checklist for analytical cross-sectional studies [28]. Mixed method studies were critically appraised using both a quantitative and qualitative approach.

### Data extraction

Key information from the included studies was extracted by two reviewers (SH and JB). The JBI’s standardized data extraction forms were used to assist this process. Extracted data were transformed to include the following details: (a) Author(s) and year of publication, (b) journal/publisher, (c) methodology, (d) setting(s), (e) characteristics of participants, (f) phenomena of interest, and (g) key findings/results.

### Data transformation, synthesis, and integration

Following the JBI’s MMSR, results and findings of the included studies were narratively synthesised using qualitative approach. Data extracted from quantitative studies were *qualitized* (i.e., statistical interpretation were transformed into narrative descriptions) [24]. Findings were systematically aggregated into categories under an umbrella category that addressed the main question of this review. This process was undertaken using NVivo12® and the meta-aggregative diagram was generated using Flowchart designer®.

## Results

### Search outcomes

The search was conducted in January 2022 using the search strings on each selected database and yielded 3,468 titles (Supplementary file 3). The identified articles were exported to Covidence® (www.covidence.org), a web-based service, where 1361 duplicate titles were removed. Two reviewers (SH and FE) manually reviewed abstracts of the remaining 2107 titles and excluded 1686 papers. The third reviewer (JB) was consulted to resolve 421 conflicting decisions between the two reviewers (SH and FE). This process left 47 articles for full-text screening and eligibility assessment (Supplementary file 4).

**Fig. 1:**
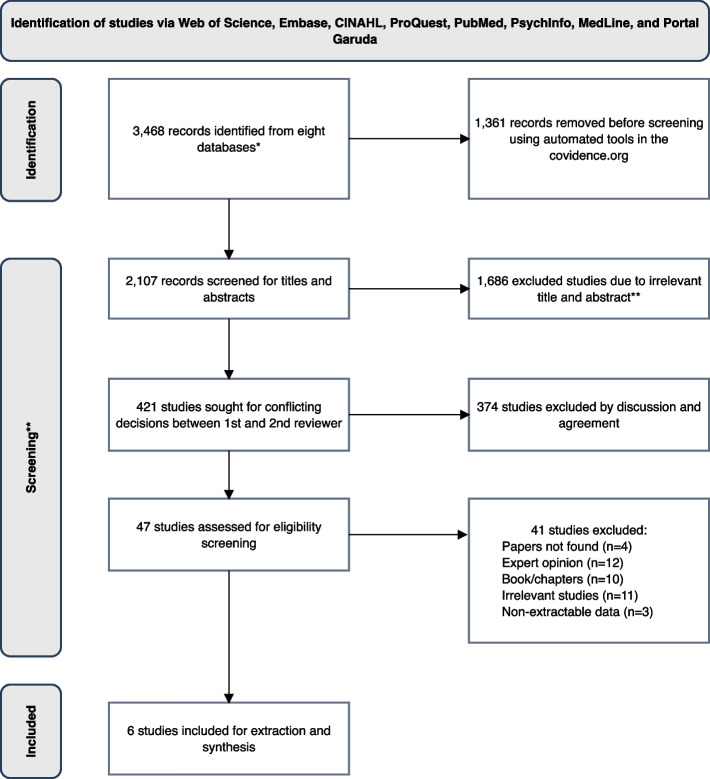
Flow diagram of the search following PRISMA (2020) [25]. *Web of Science (*n* = 927), Embase via Ovid (*n* = 621), Cumulative Index to Nursing and Allied Health Literature (CINAHL) via EBSCO (*n* = 94), ProQuest (*n* = 33), PubMed (*n* = 1295), PsychInfo via Ovid (*n* = 90), MEDLINE via Ovid (*n* = 811), and Portal Garuda (*n* = 17).**Title & abstract screening and conflict resolution were undertaken in the covidence.org

As the result of the full-text screening process (undertaken by SH and JB), 41 studies were removed (“not found” (*n* = 4), non-extractable data (*n* = 3), irrelevant type of papers (non-eligible [*n* = 12], books [*n* = 10], and opinion/discussion papers [*n* = 11])). Authors of articles classed as “not found” were contacted, but none replied. Six studies were finally included for data extraction (see Supplementary file 5). No additional papers identified from the reference list search. All of these steps were carefully recorded and reported following the 2020 PRISMA flow diagram (Fig. 1).

### Characteristics of the included studies

The six studies included in this review were conducted in The Kingdom of Saudi Arabia [29], ROC Taiwan [30], China [31], South Africa and Malawi [32], Indonesia [8], and The Netherlands [33]; within a primary care setting, including primary healthcare centres and outpatient clinics. The earliest study was published in 2009 and took place in an outpatient endocrinology clinic in Taiwan [30], and the latest was published in 2021 in Indonesia [8].

### Methodological limitations of studies

Table 3 provide a critical appraisal and assessment of the methodological quality of the included studies. This summary is based on the JBI critical appraisal checklist, with scores for the included studies ranging from 5/10 to 9/10. These scores reflect how well each study has addressed possibility of bias in its design, conduct and analysis [34]. This review did not exclude studies with low scores. To gather the information of this review, it considered each study and its contribution to the overall body of knowledge.

**Table 3:** Critical appraisal results for included studies using the JBI Critical Appraisal Checklist^a^

Authors (year)	Study design	Q1	Q2	Q3	Q4	Q5	Q6	Q7	Q8	Q9	Q10	Total
Almigbal and Schattner (2018) [35]	Cross-sectional	Y	Y	Y	Y	N	N	Y	Y	N/A	N/A	6/8
Jiann, et al. (2009) [30]	Cross-sectional	Y	Y	Y	Y	N	N	Y	Y	N/A	N/A	6/8
Lo, et al. (2014) [31]	Cross-sectional	Y	Y	Y	Y	Y	Y	Y	Y	N/A	N/A	8/8
Cooper, et al. (2018) [36]	Qualitative	U	Y	U	U	U	Y	N	Y	Y	Y	5/10
Hadisuyatmana, et al. (2021) [8]	Qualitative	Y	Y	Y	Y	Y	N	N	Y	Y	Y	8/10
Rutte, et al. (2016) [33]	Quantitative component of Mixed-method study	N	N	U	N	Y	Y	Y	Y	Y	N/A	5/9
Rutte, et al. (2016) [33]	Qualitative component of Mixed-method study	Y	Y	Y	Y	N	Y	Y	Y	Y	Y	9/10

*legend*: *Q#* question number, *Y* yes, *N* no, *U* unclear, *N/A* not applicable

^a^All included articles were appraised using the appropriate critical appraisal checklist sourced from the Joanna Briggs Institute. Qualitative studies were appraised using a critical appraisal checklist for qualitative research, cross-sectional studies were assessed using the appraisal checklist for analytical cross-sectional studies, and the Mixed method study components were separately appraised using quantitative and qualitative appraisal checklists [27, 28]

### Focus of the included studies

All studies involved men with T2DM as the research participants and source of data for analysis. While the focus of each study varied, there were overlaps between the various designs. The determinants around the ‘willingness to discuss ED in men with T2DM’ were found in studies conducted in the Kingdom of Saudi Arabia [29], ROC Taiwan [30], and Hong Kong [31]. Studies originating from South Africa [32], Indonesia [8], and the Netherlands [33] highlighted the personal experiences of men with T2DMED in seeking help. In addition, studies from Hong Kong [31], Indonesia [8], and the Netherlands [33] outline the expectations, needs and preferences of T2DMED men towards the levels of professional help they expected to receive. A total of 2111 men participated in the included studies (see Table 4), with key findings presented in Table 5.

**Table 4:** Characteristics of the included study following the Joanna Briggs’ Institute’s data extraction table

**Authors, year**	**Settings, context-related information**	**Duration/year of data collection**	**Methods, type of study**	**Characteristics of participants**	**Phenomena of interest/**research aim
Almigbal and Schattner (2018) [29]	Primary care clinics, Kingdom of Saudi Arabia	July–September, 2015	Cross-sectionals, Quantitative	(*n*) 309 Saudi Arabian men (60.2 years old avg.), of which mostly had been living with diabetes up to 12.5 years	Investigating on the proportion of Saudi men with T2DM who have been asked about ED by their physicians and the determinants around the willingness of the men to discuss ED
Jiann et al. (2009) [30]	Outpatient endocrinology clinic, Taiwan	June 2006–May 2007	Cross-sectionals, Quantitative	916 Taiwan-Chinese men (26–85 years old) who lived with T2DM	Evaluating the prevalence of ED in T2DM patients, their treatment-seeking patterns, and factors affecting them
Lo et al. (2014) [31]	Government outpatient clinics, Hong Kong, China	May 2012	Cross-sectionals, Quantitative	603 Chinese-Hong Kong men aged 60.5 ± 10.5 years	Identifying the prevalence of ED and the factors associated with T2DM patients’ attitude toward ED and their expectation for ED management in the primary care setting
Cooper et al. (2018) [32]	Personal and group appointments, South Africa and Malawi	November 2015–July 2017	Qualitative	47 South African and Malawian men aged 28–78 years old	Exploring the relationship between the biomedical and social experiences of sexuality; and gaining understandings the distinction between experiences of sexual functioning and sexual well- being in men with T2DM
Hadisuyatmana et al. (2021) [8]	Urban area of Surabaya, (Indonesia)	September–December 2018	Descriptive qualitative	12 Indonesian men aged between 45- and 53 years old living with T2DM up to 13 years	Exploring how sexual relationships are experienced and lived by men with T2DM in Indonesia, as well as how care was received from the primary health services
Rutte et al.(2016) [33]	Primary and secondary diabetes care centres, the Netherlands	The detail is not available	Cross-sectional and individual interview, mixed-method	107 men who were diagnosed with T2DM	Exploring the needs and preferences for care concerning sexual problems in both men and women with T2DM

**Table 5:** Key findings reported in the included study following data extraction

*Authors*	*Key findings*
Almigbal and Schattner (2018) [29]	Only few men had been questioned by their physicians about ED, despite the expectation expressed by most of them The participants who complained of severe ED or were older than 60 were unwilling to discuss ED. "Embarrassing the doctor", "ED is a personal issue", "too old to address ED issues ", "feeling embarrassed to talk about it", "too sick now to address ED issues", "no effective treatment is available", and "my doctor is too young to discuss my ED” were reported as the barriers for the men to seek help
Jiann et al. (2009) [30]	The majority of the men suffered from severe ED. Less than a third of them had ever sought treatment for ED. Embarrassment and misinformation about ED treatment were the main causes for not seeking professional help. Most men wanted their doctors to initiate discussion of ED
Lo et al. (2014) [31]	Only half of the men participating in the study were aware of ED. Amongst those who have ED, only a third viewed ED as an illness that requires treatment or as a consequence of an illness. Few of these men had ever sought help from any doctor, although most participants expected help
Cooper et al. (2018) [32]	Sexual difficulties emerged as a key and pressing concern for men with diabetes in this study. Instead of receiving supports, most men in the study reported the dismissive and punitive responses expressed by the HCP as the drive to avoid seeking help
Hadisuyatmana et al.(2021) [8]	Sense of embarrassment, perceiving the doctor would not have enough time to consult, and lack of knowledge have become the barriers for the men from raising questions around ED with the HCP. Although, the men were expecting help and discussions initiated by the HCP
Rutte et al. (2016) [33]	This study identified that most of the men were aware of sexual dysfunction associated with diabetes (DSD). Many of them had needed help, and some had contacted a care provider for sexual problems. These few men were all dissatisfied with the offered care. Some HCP who received their request remained silence and did not offer any assistance, and some other HCP seemed lacking knowledge. Meanwhile, the men had the impression that HCP were embarrassed, not capable, insecure, or did not want to be burdened with such a discussion. A few patients who were provided with phosphodiesterase-5 inhibitors often found that the medication did not help. Instead, they would prefer a psychological help. However, GPs were often thought to lack time for DSD discussion. Other patients preferred the diabetes nurse, since she is responsible for diabetes care, but some patients doubted the knowledge of diabetes nurses on sexuality

### Meta-aggregative map and categories of findings

**Fig. 2:**
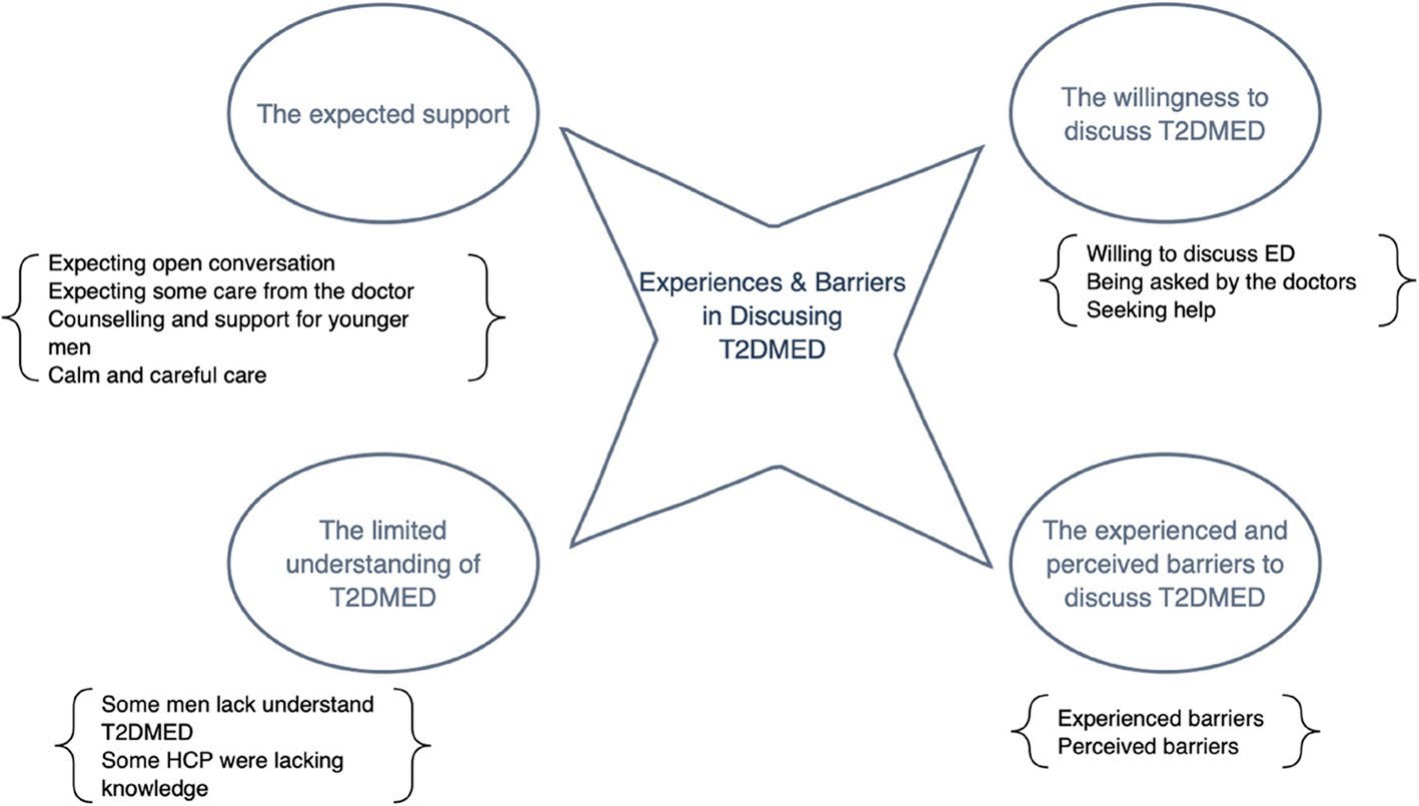
Meta-aggregative map generated from the included studies

Following JBI’s MMSR, illustrations, findings, and categories were synthesized inductively while maintaining a focus on the pre-determined objectives of the review [24]. Using NVivo 12®, a meta-aggregative map and codebook of findings were generated to identify initial categories. The categories of the identified findings were constructed using a meta-aggregative map to illustrate the four main categories (which cover thirteen findings) (Fig. 2), including: (1) the willingness to discuss T2DMED, (2) the experienced and perceived barriers, (3) the limited understanding of T2DMED, and (4) the expected support.

### The willingness to discuss T2DMED

The included studies shared different findings with regard to the men’s willingness to discuss ED with their care providers. While most men were reportedly willing to discuss ED, only a few of them have ever been asked about ED by their HCPs. Such findings were reported in Almigbal and Schattner [29] and Jiann, et al. [30]. Almigbal and Schattner [29] interpret their study findings as.

“Among the respondents who have not yet been asked about ED in the last year by their physicians, most (91%) of them have ED and would be willing to discuss it with their physicians (*P* = 0.02). Even if they do not have ED, twice as many are willing to discuss this matter as unwilling.”

“Few (9.7%) patients with type 2 diabetes mellitus have been asked about ED in the past year by their physicians.”

Jiann, et al. [30] added that a similar phenomenon occurred in their study:

“Of all the subjects (men) with ED (701), more than half (56.6%) wished to discuss ED with their doctors. Of all the respondents, most (90.4%) wanted their doctors to initiate discussion.”

“Only a small number (7.9% [71/899]) of the sample group had been asked about this subject by their doctors.”

However, some other men were reported to have reached out to their HCP for some help. This finding was found in a mixed-method study by Rutte, et al. [33] that explored the phenomenon in the Netherlands, and suggested that only a few men had ever sought help for T2DMED.

*“Furthermore, less than half (41%) of these men had ever contacted a care provider.”* Rutte, et al. [33]

### Experienced and perceived barriers

This category covers experienced and perceived barriers relating to discussing T2DMED reported in the included studies.

#### Experienced barriers

Economic burden and negative responses received from HCPs in the past have been identified as barriers to the men raising questions about ED. Economic burden and the potential cost of the medication for ED are reported as constraints that discouraged men from raising their sexual problems. This finding was found in four studies [29–32]. The authors also found that ED discussions or therapies were less desired by those with a low monthly income.

Almigbal and Schattner [29] interpret their findings:

“Participants with low monthly incomes (i.e., < 5000 SR, by 53.2% participants) were unwilling to discuss ED with their physicians (*P* = 0.03).”

Cooper, et al. [32] commented:

“Lack of affordability was another obstacle, as these drugs are costly.”

Jiann, et al. [30] reasoned:

“Economic burden reasoned some men to avoid consulting their ED to physician.”

Lo, et al. [31] suggested:

“The acceptance of drug therapy for ED was relatively low in our subjects (16.9%). The cost of PDE-5 is likely a concern.”

In some cases, the negative responses received from the HCPs were reported as cause of the men’s reluctance to seek help from ED. Neglect, punitive response and a feeling of bulling were the responses expressed by men in a study conducted in sub-Saharan countries of Africa by Cooper, et al. [32] and in a mixed-method study in the Netherlands by Rutte, et al. [33]. These findings suggest that the negative experiences were not only exclusive to communities in less-developed countries but also occur in a well-developed country.

Cooper, et al. [32] quoted:

“Most had not raised it, and the few who had, were unsatisfied with the responses they received. They reported that healthcare providers had never asked about or offered any information on sexual functioning at routine follow-up visits.”

“I’ve shared my problem with them. But. . .the nurse, she just said ‘Look at your health. You know why the boy down there won't work. You don’t look after yourself. If you started to take care of your body, your problem would go away.”

“I don’t say anything. You know how they are at the clinic. . .They shout at you when you ask things. Tell you to stop complaining. And sometimes even punish you for wasting time. . .So, I’m scared to speak to the doctor.”

Under the same shade, Rutte, et al. [33] added:

“Some patients had sought help for their sexual problems, but they were all dissatisfied with the offered care. Some care providers took the question of the patient for granted and provided no further help.”

#### Perceived barriers

Issues of embarrassment were consistently identified as barriers expressed by men in most of the included studies. For many men, ED was regarded as a personal issue and asking about it was considered embarrassing both for themselves and for the doctors.

As interpreted by Almigbal and Schattner [29]:

*“*ED is a personal issue for many men (60.6%). It is a personal issue.”

“The main obstacles to discussing ED with the doctors is: embarrassing my doctor (63.9%, *P* < 0.001).”

Cooper, et al. [32] argued:

“Reasons for not raising the topic with healthcare providers included embarrassment and feeling ‘awkward’ or ‘uncomfortable’ about discussing the issue.”

Hadisuyatmana, et al. [8] quoted their 9th participant:

“I don’t want to ask this (erectile dysfunction) to any doctor…I am too ashamed about this, so I never asked this to anyone.”

Jiann, et al. [30] concluded that:

“Many (42.8%) men (felt) embarrassed to talk about it.”

Rutte, et al. [33] quoted their 17th participant’s statement:

“I wouldn’t dare [...] I feel embarrassed about it.”

For some men with T2DM, the reluctance to address ED also emerged from an assumption that the HCP was too busy and did not have enough time for discussions. As a result, the men deliberately chose not to ask about their problem.

Hadisuyatmana, et al. [8] quoted their 8th participant’s:

“The Puskesmas was always busy, there will not be sufficient time for us to consult with the doctor.”

Participant 19 in Rutte, et al. [33] added:

“I think that they [care providers] think: I don’t want to be burdened with their problems.”

The tendency to avoid discussing ED was more profound in men older than 60 years of age and those with more severe ED. These men reasoned that it was too late to start asking about ED due to the advancing age and progressing disease.

Almigbal and Schattner [29] suggested that

“The patients above 60 years were mostly (70%) less willing to discuss ED with their physicians compared to the patients less than 60 years old. Too old now (59.4%, P < 0.001).”

“… among participants who have ED, those who were complaining of severe ED (63.1) were unwilling to discuss it with their physicians.”

“The level of ED severity plays a major role, with this study showing that patients who have diabetes with severe ED are less willing to discuss this with their physicians compared to those with mild ED.”

A few men were reportedly seeking help despite their severe ED. For instance, a study involving Chinese population Lo, et al. [31] identified that.

“Very few (<10%) of the subjects with ED had ever sought help from any doctor regardless of degree of severity.”

Nonetheless, most participating men believed that there was nothing that could be done to regain erectile function. The men were disappointed if the medication received to control T2DM did not work. As a result, many of these men chose to ignore and bury the problem. As Cooper, et al. [32], Almigbal and Schattner [29], and Rutte, et al. [33] quoted:

Interviewed participant in Cooper, et al. [32]:

“I’ve come to realise that I must just accept it, ‘cause nothing can be done. No-one can help me.”

Rutte, et al. [33] quoted their participant 17:

“I went to the doctor, who gave me two pills . . . no effect, never talked about it again.”

Almigbal and Schattner [29] concluded that

“Many men believe that there is no effective treatment available (54.8%, *P* < 0.001).”

### Limited understanding of T2DMED

Lack of understanding of T2DMED is identified as another contributor to the absence of discussion during consultations. The findings of the included studies indicate that most men did not comprehend the association between ED and their T2DM diagnosis. Instead, they recognized ED as a normal consequence of ageing. The study by Lo, et al. [31] concluded that:

“Only less than a third (30%) of the respondents in this study regarded ED as a disease which requires treatment, and almost half (45%) of them thought it was simply a consequence of aging.”

Lack of knowledge by HCPs was also reported as a factor that resulted in men not getting additional help. Avoidance and dismissive attitudes of the men were suggested as an indication that the HCPs were lacking knowledge that resulted in them not offering help. The study conducted in the Netherlands, Rutte, et al. [33] supported:

“Other care providers seemed not able to talk about it or were lacking knowledge.”

In another study, Cooper, et al. [32] reported that the men were often dismissed by the HCPs and assumed that ED was normal and should be accepted as the consequence of the ageing process:

“Healthcare providers reportedly often dismissed their concerns by saying that sexual dysfunction was normal with ageing and that the men should just accept this.”

### The expected support

All studies reported that the men were expecting some support to recover their erectile function. Four types of support were identified in this review including conversation initiatives, ED management, counselling, and empathetic care. Most participating men expected the doctors to initiate open conversations to alleviate their hesitancy from asking about sexual concerns. Rutte, et al. [33] responded to their participants’ voices:

“Patients mentioned that if they had been informed, that would enable them to start a discussion with their care provider more easily because it would take away their feeling that a sexual dysfunction is an unusual problem.”

“Moreover, they felt that if the care provider would bring up this topic (ED), it might lower the threshold for discussion. It would help if the care provider had an open attitude about sexuality.”

Likewise, Hadisuyatmana, et al. [8] quoted their participants concern:

“I actually wished for interactions. But they (health professionals) sometimes didn’t have the initiative to ask, and we don’t start the talk, and just kept quiet.”

The men also expected some sort of ED management from the doctors who diagnosed their T2DM, whom the men respected as a reliable source of help. Lo, et al. [31] confirmed this finding:

“Most (76.1%) of the subjects preferred receiving management from doctors should they be diagnosed with ED.”

The men expected to have counselling sessions that allowed them to raise their concerns around T2DMED. One participant underlined that this kind of support would be expected by men of younger age and those who had just started to live as a couple. Rutte, et al. [33] and Hadisuyatmana, et al. [8] informed the preferred form of care as.

Authors’ interpretation in Rutte, et al. [33]:

The preferred form of care were emotional care, medical care, information, and to be contacted by a caregiver for sexual issues [33].

Participant 8 in Hadisuyatmana, et al. [8]:

There should be a counsellor for sexual things […] I believe there are many patients who would like to ask questions […] They might want to fix things […] there’s nothing wrong with trying to do that […] especially for those who are younger than 40…those who just started to live as a family (married couple).

Finally, despite the limited time for service, the men expected the care to be delivered with empathy. Participant 6 in Hadisuyatmana, et al. [8] expressed:

“I expect that they are not rushing when caring for people…despite they are busy…it is all about time management. What is important that all patients should be taken in a good care, that is the point. Not in a rush they need to care for us with heart.”

## Discussion

This systematic review was developed to identify the experiences, facilitators, and barriers around T2DMED screening and discussions reported by men and HCPs. Quantitative, qualitative, and mixed-method studies of relevant topics were critically appraised and synthesized to better understand T2DMED. In the end, six papers published within the last two decades were included in the review. From the included studies, it was observed that no HCPs were identified as participants in any of the studies. This indicates that there was limited primary evidence available around the selected topic from HCPs perspectives, suggesting a priority in future research.

The small number of included studies in this systematic review reflect a paucity of research addressing T2DMED related experiences and barriers. An earlier systematic review by Williams et al. [26] looking at the factors influencing ED treatment in the general population included 50 studies for extraction. However, in the current review, only limited number of studies were found that have investigated T2DMED associated barriers and experiences, indicating a need for further research in this area.

This present review shares similarities to findings from an earlier systematic review by Teo, et al. [17], in which the authors investigated factors that influence men’s health screening uptake and individual, social, and health system domain factors that influenced ED treatment. In that review, knowledge, service cost, and health professionals’ attitudes were highlighted as some of the factors associated with men’s access to health screening services. The present review did not undertake similar analytical procedures due to the limited studies included for extraction. Rather, it extends knowledge of these factors related to the willingness to discuss T2DMED, the perceived barriers, and in what ways help is expected by the affected men.

The overall findings of the review indicate that T2DMED screening and associated discussions were expected by most men. In reality, a minority of the men had been asked about ED by their physicians. This is an important finding as very few of the men had ever proactively sought professional help for their ED. This unmet expectation has been discussed elsewhere [18, 37] and suggests that some men were reluctant to proactively seeking help as it may impact their masculine identity [38, 39].

The reluctance of HCPs to raise ED as a topic for discussion with male patients had been discussed in an editorial letter [18]. In the letter, Tisdall, et al. [18] outlined an expectation by HCPs that men should raise ED as a topic for discussion, perceiving ED as an appropriate topic for men to raise at a consultation [18]. Nonetheless, this view was not universal as a few HCPs (i.e., doctors) reportedly addressed ED with their patients during appointments [29].

The findings of this review indicate that the initiation of T2DMED discussions might be impacted by multiple barriers. Concerns associated with embarrassment around discussing ED are coupled with the cost of medical treatments; often reducing efforts to regain erectile function. The lack of knowledge about T2DMED often leads to a misconception that ED is simply a consequence of the ageing process. This finding suggests that efforts to alleviate embarrassment and improved education can help men better understand T2DM and realise the significant benefits of well-managed diabetes. In a general population, an earlier study suggested that education was significant in improving men’s intention to self-report and the early screening of ED [40]. Rushforth, et al. [41] suggest that knowledgeable patients obtain sufficient skills to manage their underlying disease and control comorbidities. Therefore, alleviating embarrassment and patient education are important initiatives to improve T2DMED [42].

The sense of despair expressed by some men in the included studies due to negative experiences with the HCPs is concerning [29, 30, 32] HCPs are often under time constraints, which may affect their willingness to initiate discussions with patients. This, along with their attitudes, has been identified as a significant barrier to patients seeking professional help [8, 32, 33]. This shows that there is a requirement for ongoing training and development to enable HCPs to effectively deal with T2DMED.

The messages gathered from four studies in this review underline the support and assistance expected by men [8, 30, 31, 33]. These include initiatives that help open discussions, ED management, counselling, and education. Studies by Hadisuyatmana, et al. [8], Jiann, et al. [30], and Rutte, et al. [33] highlighted men’s expectation that doctors would open interactions around T2DMED. Rutte, et al. [33] suggested that this will lower barriers and allow the men to ask more questions about their ED.

In terms of the point of interaction, GPs were preferred by most men as a reliable source to help them cope with T2DMED [31, 33] For this reason, efforts to empower care providers’ and create awareness of T2DMED are important [43] and the integration of care for diabetes into primary care services is inevitable [41]. Nonetheless, the absence of HCPs as participants in this present review makes it difficult to draw any clear conclusions. Their participation in future research is paramount to narrow the gap in the current body of knowledge. The types of help that were identified as needed by men were emotional support, medical care, and consultations [8, 33]. Further research is important to determine the appropriate support and care for men with T2DMED in the future [8].

## Limitations

There are limitations associated with this systematic review. The strict criteria and the use of sub-headings for inclusions might have resulted in some studies being overlooked. The limitation to only include peer-reviewed studies might have underestimated the possible findings of grey literature. However, the inclusion of eight prominent health and related science databases is believed to be comprehensive. The small number of included studies in this review informs the knowledge gap and highlights the areas for future investigations.

By nature, systematic reviews do not offer novel findings. Rather, they synthesize evidence found from earlier studies, inform gaps in the research, and substantiate the evidence as a guide to direct future policy, practice, and research. In addition, conclusions drawn from MMSR are generated through narrative synthesis and the discussion is directed to establish a line of argument based on the findings of the reviewed topic [24]. Hence, they are incomparable with meta-analysis and limitedly generalizable.

The research included in this review all had similar yet distinct areas of focus, which collectively helped to address the aims of the study. It appears that there is a gap in knowledge regarding the involvement of health professionals in the screening, discussing, and managing of T2DMED. This knowledge gap highlights the need for further exploration into the barriers and facilitators associated with these practices, in order to improve policy and practice in the future.

## Conclusion

During health appointments, screening and discussion regarding T2DMED are often reported as rare. This review primarily aims to investigate the barriers and experiences surrounding this issue. This review concludes that T2DMED was rarely addressed with the absence of HCP participation the included studies. Embarrassment, the cost of potential treatments, lack of knowledge, and the negative attitudes of HCP are identified as the barriers to T2DMED discussions reported by the patients. Emotional and medical care are essential forms of assistance that are expected by the patients. This review strongly the need to empower healthcare professionals to address ED in men living with T2DM.

### Recommendations for practice

This review unveiled that the absence of T2DMED discussion is associated with the patient’s lack of knowledge and HCPs’ lack of understanding of the topic, as well as a hesitancy to initiate the discussion. Despite this, the urgency to mitigate future health consequences suggests a need to break down the identified barriers and promote T2DMED screening and discussion. With regard to the identified barriers, a more T2DMED-friendly environment and training for the HCPs should be seen as potential solutions to promote the need for T2DMED screening uptake. In addition, education that fits the men’s unique characteristics and designed to improve understanding of the issue is needed.

### Recommendations for research

The present review was unable to identify and include studies that involved HCPs. Therefore, this review only reported on male patients’ experiences with HCPs (i.e., doctors and nurses). This suggests further exploration from HCPs perspectives around recommended practices for T2DMED.

### Supplementary Information

**Additional file 1.** PRISMA 2020 Checklist.

**Additional file 2.** JBI Critical Appraisal Tools.

**Additional file 3.** Search strings and history logs.

**Additional file 4.** Eligibility assessment.

**Additional file 5.** JBI's data extraction forms.

## Declarations

### Competing of interests

All authors declare no conflicting personal or professional interests that may influence the work reported in this review.
