# Supplementary material for: The experiences and barriers in addressing type 2 diabetes mellitus-associated erectile dysfunction: a mixed method systematic review
Source: Syst Rev. 2023 Aug 10;12:138. doi: 10.1186/s13643-023-02303-4 (PMC10416416; doi:10.1186/s13643-023-02303-4)
Supplement: Supplementary file 2 — Additional file 2. JBI Critical Appraisal Tools. [file 13643_2023_2303_MOESM2_ESM.pdf]

Additional File 1: JBI Critical Appraisal Checklist For Qualitative Research

Reviewer____Date________________________________

Author____Year_________ Record Number_________

		Yes	No	Unclear	Not applicable
1.	Is there congruity between the stated philosophical perspective and the research methodology?	☐	☐	☐	☐
2.	Is there congruity between the research methodology and the research question or objectives?	☐	☐	☐	☐
3.	Is there congruity between the research methodology and the methods used to collect data?	☐	☐	☐	☐
4.	Is there congruity between the research methodology and the representation and analysis of data?	☐	☐	☐	☐
5.	Is there congruity between the research methodology and the interpretation of results?	☐	☐	☐	☐
6.	Is there a statement locating the researcher culturally or theoretically?	☐	☐	☐	☐
7.	Is the influence of the researcher on the research, and vice- versa, addressed?	☐	☐	☐	☐
8.	Are participants, and their voices, adequately represented?	☐	☐	☐	☐
9.	Is the research ethical according to current criteria or, for recent studies, and is there evidence of ethical approval by an appropriate body?	☐	☐	☐	☐
10.	Do the conclusions drawn in the research report flow from the analysis, or interpretation, of the data?	☐	☐	☐	☐

Overall appraisal: Include ☐ Exclude ☐ Seek further info ☐

Comments (Including reason for exclusion)

____

____

Additional file 2

JBI Critical Appraisal Checklist for Studies Reporting Prevalence Data

Reviewer____Date________________________________

Author____Year_________ Record Number_________

		Yes	No	Unclear	Not applicable
1.	Was the sample frame appropriate to address the target population?	□	□	□	□
2.	Were study participants sampled in an appropriate way?	□	□	□	□
3.	Was the sample size adequate?	□	□	□	□
4.	Were the study subjects and the setting described in detail?	□	□	□	□
5.	Was the data analysis conducted with sufficient coverage of the identified sample?	□	□	□	□
6.	Were valid methods used for the identification of the condition?	□	□	□	□
7.	Was the condition measured in a standard, reliable way for all participants?	□	□	□	□
8.	Was there appropriate statistical analysis?	□	□	□	□
9.	Was the response rate adequate, and if not, was the low response rate managed appropriately?	□	□	□	□

Overall appraisal: Include □ Exclude □ Seek further info □

Comments (Including reason for exclusion)

____

____

JBI Critical Appraisal Checklist for Case Series

Reviewer______________________________________Date______________________________

Author_______________________________________Year_________ Record Number_________

	Yes	No	Unclear	Not applicable
• Were there clear criteria for inclusion in the case series?	☐	☐	☐	☐
• Was the condition measured in a standard, reliable way for all participants included in the case series?	☐	☐	☐	☐
• Were valid methods used for identification of the condition for all participants included in the case series?	☐	☐	☐	☐
• Did the case series have consecutive inclusion of participants?	☐	☐	☐	☐
• Did the case series have complete inclusion of participants?	☐	☐	☐	☐
• Was there clear reporting of the demographics of the participants in the study?	☐	☐	☐	☐
• Was there clear reporting of clinical information of the participants?	☐	☐	☐	☐
• Were the outcomes or follow up results of cases clearly reported?	☐	☐	☐	☐
• Was there clear reporting of the presenting site(s)/clinic(s) demographic information?	☐	☐	☐	☐
• Was statistical analysis appropriate?	☐	☐	☐	☐

Overall appraisal: Include ☐ Exclude ☐ Seek further info ☐

Comments (Including reason for exclusion)

____

____

© JBI, 2020. All rights reserved. JBI grants use of these tools for research purposes only. All other enquiries should be sent to jbisynthesis@adelaide.edu.au.

JBI Critical Appraisal Checklist for Analytical Cross Sectional Studies

Reviewer____Date________________________________

Author____Year_________ Record Number_________

	Yes	No	Unclear	Not applicable
1. Were the criteria for inclusion in the sample clearly defined?	□	□	□	□
2. Were the study subjects and the setting described in detail?	□	□	□	□
3. Was the exposure measured in a valid and reliable way?	□	□	□	□
4. Were objective, standard criteria used for measurement of the condition?	□	□	□	□
5. Were confounding factors identified?	□	□	□	□
6. Were strategies to deal with confounding factors stated?	□	□	□	□
7. Were the outcomes measured in a valid and reliable way?	□	□	□	□
8. Was appropriate statistical analysis used?	□	□	□	□

Overall appraisal: Include □ Exclude □ Seek further info □

Comments (Including reason for exclusion)

____

____

____

© JBI, 2020. All rights reserved. JBI grants use of these tools for research purposes only. All other enquiries should be sent to jbisynthesis@adelaide.edu.au.
