# Supplementary material for: The experiences and barriers in addressing type 2 diabetes mellitus-associated erectile dysfunction: a mixed method systematic review
Source: Syst Rev. 2023 Aug 10;12:138. doi: 10.1186/s13643-023-02303-4 (PMC10416416; doi:10.1186/s13643-023-02303-4)
Supplement: Supplementary file 3 — Additional file 3. Search strings and history logs. [file 13643_2023_2303_MOESM3_ESM.docx]

Supplementary file 3. Search strings used and count of identified titles.

### #1 Database: Web of Science

| # | **Filters** | **Filters: Full-text, English, Bahasa Indonesia, year 2001-current, NOT: review**, **book, report, single case, documents, opinion.** | **n** |
| --- | --- | --- | --- |
| 1 | Concept 1: erectile dysfunction | TS=(‘Erectile Dysfunction’ OR ‘erectile disfunction’ OR ‘impotence’ OR ‘impotent’ OR ‘male impotence’ OR ‘male impotent’ OR ‘male sexual impotence’ OR ‘male sexual impotent’ OR ‘male sexual dysfunction’ OR ‘male sexual disfunction’) and Articles (Document Types) and Male or Humans or Erectile Dysfunction (MeSH Headings) and English or Indonesian (Languages) | 24,327 |
| 2 | Concept 2: diabetes mellitus | TS=(‘diabetes mellitus’ OR ‘type-2 diabetes mellitus’ OR ‘diabetes mellitus type-2’ OR ‘type-2 diabetes’ OR DM OR ‘hyperglycaemia’ OR ‘adult onset diabetes’) and Articles (Document Types) and Humans or Male or Diabetes Mellitus Type 2 (MeSH Headings) and English or Indonesian (Languages) | 359,180 |
| 3 | Concept 3: screening | TS=(‘screen’ OR ‘screening’ OR ‘discuss’ OR ‘discussion’ OR ‘diagnose’ OR ‘diagnosis’ OR ‘management’ OR ‘manage’) and Articles (Document Types) and Humans or Male (MeSH Headings) and English (Languages) | 3,892,996 |
| 4 | Concept 4: barriers | (TI=(experienc* OR barrier OR barriers OR challeng* OR facilitat* OR determinan OR determinants OR treatment OR intervention)) AND (DT==("ARTICLE") AND MH==("HUMANS" OR "MALE")) | 1,944,659 |
| 5 | Search #5 | #1 AND #2 | 2,312 |
| 6 | Search #6 | #3 OR #4 | 3,892,996 |
| 7 | Search #7 | #5 AND #6 [(#1 AND #2) AND (#3 OR #4)] | 927 |

### #2 Database: Embase via Ovid

| # | **Filters** | **Filters: Full-text, English, Bahasa Indonesia, year 2001-current, NOT: review**, **book, report, single case, documents, opinion.** | **n** |
| --- | --- | --- | --- |
| 1 | Concept 1: erectile dysfunction | (Erectile Dysfunction or erectile disfunction or impotence or impotent or male impotence or male impotent or male sexual impotence or male sexual impotent or male sexual dysfunction or male sexual disfunction).mp. [mp=title, abstract, heading word, drug trade name, original title, device manufacturer, drug manufacturer, device trade name, keyword heading word, floating subheading word, candidate term word]  limit to (full text and human and yr="2001 - 2021") | 4,344 |
| 2 | Concept 2: diabetes mellitus | (diabetes mellitus or type-2 diabetes mellitus or diabetes mellitus type-2 or type-2 diabetes or DM or hyperglycaemia or adult onset diabetes).mp. [mp=title, abstract, heading word, drug trade name, original title, device manufacturer, drug manufacturer, device trade name, keyword heading word, floating subheading word, candidate term word]  limit to (full text and human and yr="2001 - 2021") | 123,464 |
| 3 | Concept 3: screening | (screen or screening or discuss or discussion or diagnose or diagnosis or management or manage).mp. [mp=title, abstract, heading word, drug trade name, original title, device manufacturer, drug manufacturer, device trade name, keyword heading word, floating subheading word, candidate term word]  limit to (full text and human and yr="2001 - 2021") | 736,363 |
| 4 | Concept 4: barriers | (experienc* or barrier or barriers or challeng* or facilitat* or determinan or determinants or treatment or intervention).mp. [mp=title, abstract, heading word, drug trade name, original title, device manufacturer, drug manufacturer, device trade name, keyword heading word, floating subheading word, candidate term word]  limit to (full text and human and yr="2001 - 2021") | 914,177 |
| 5 | Search #5 | #1 AND #2 | **814** |
| 6 | Search #6 | #3 OR #4 | **1,282,466** |
| 7 | Search #7 | #5 AND #6 [(#1 AND #2) AND (#3 OR #4)] | **621** |

### #3 Database: CINAHL via EBSCO

| # | **Filters** | **Filters: Full-text, English, Bahasa Indonesia, year 2001-current, NOT: review**, **book, report, single case, documents, opinion.** | **n** |
| --- | --- | --- | --- |
| 1 | Concept 1: erectile dysfunction | ‘Erectile Dysfunction’ OR ‘erectile disfunction’ OR ‘impotence’ OR ‘impotent’ OR ‘male impotence’ OR ‘male impotent’ OR ‘male sexual impotence’ OR ‘male sexual impotent’ OR ‘male sexual dysfunction’ OR ‘male sexual disfunction’  **Limiters** - Peer Reviewed; English Language; Published Date: 20010101-20211231; Research Article; Human; Sex: Male  **Expanders** - Apply equivalent subjects  **Search modes** - Boolean/Phrase | 1,819 |
| 2 | Concept 2: diabetes mellitus | ‘diabetes mellitus’ OR ‘type-2 diabetes mellitus’ OR ‘diabetes mellitus type-2’ OR ‘type-2 diabetes’ OR DM OR ‘hyperglycaemia’ OR ‘adult onset diabetes’  **Limiters** - Peer Reviewed; English Language; Published Date: 20010101-20211231; Research Article; Human; Sex: Male  **Expanders** - Apply equivalent subjects  **Search modes** - Boolean/Phrase | 50,333 |
| 3 | Concept 3: screening | ‘screen’ OR ‘screening’ OR ‘discuss’ OR ‘discussion’ OR ‘diagnose’ OR ‘diagnosis’ OR ‘management’ OR ‘manage’  **Limiters** - Peer Reviewed; English Language; Published Date: 20010101-20211231; Research Article; Human; Sex: Male  **Expanders** - Apply equivalent subjects  **Search modes** - Boolean/Phrase | 330,982 |
| 4 | Concept 4: barriers | experienc* OR barrier OR barriers OR challeng* OR facilitat* OR determinan OR determinants OR treatment OR intervention  **Limiters** - Peer Reviewed; English Language; Published Date: 20010101-20211231; Research Article; Human; Sex: Male  **Expanders** - Apply equivalent subjects  **Search modes** - Boolean/Phrase | 514,426 |
| 5 | Search #5 | #1 AND #2 | **153** |
| 6 | Search #6 | #3 OR #4 | **681,308** |
| 7 | Search #7 | #5 AND #6 [(#1 AND #2) AND (#3 OR #4)] | **94** |

### #4 Database: PubMed

| # | **Filters** | **Filters: Full-text, English, Bahasa Indonesia, year 2001-current, NOT: review**, **book, report, single case, documents, opinion.** | **n** |
| --- | --- | --- | --- |
| 1 | Concept 1: erectile dysfunction | 'Erectile Dysfunction' OR 'erectile dysfunction' OR 'impotence' OR 'impotent' OR 'male impotence' OR 'male impotent' OR 'male sexual impotence' OR 'male sexual impotence' OR 'male sexual dysfunction' OR 'male sexual dysfunction'  *Filters applied:*Humans, English, Indonesian, Male, from 2001 - 2021*.* | 18,996 |
| 2 | Concept 2: diabetes mellitus | ‘diabetes mellitus’ OR ‘type-2 diabetes mellitus’ OR ‘diabetes mellitus type-2’ OR ‘type-2 diabetes’ OR DM OR ‘hyperglycaemia’ OR ‘adult onset diabetes’  *Filters applied:*Humans, English, Indonesian, Male, from 2001 - 2021*.* | 175,312 |
| 3 | Concept 3: screening | ‘screen’ OR ‘screening’ OR ‘discuss’ OR ‘discussion’ OR ‘diagnose’ OR ‘diagnosis’ OR ‘management’ OR ‘manage’  *Filters applied:*Humans, English, Indonesian, Male, from 2001 - 2021*.* | 3,121,962 |
| 4 | Concept 4: barriers | experienc** OR barrier OR barriers OR challeng** OR facilitat** OR determinan OR determinants OR treatment OR intervention  *Filters applied:*Humans, English, Indonesian, Male, from 2001 - 2021*.* | 3,906,970 |
| 5 | Search #5 | #1 AND #2 | 1,401 |
| 6 | Search #6 | #3 OR #4 | 4,172,832 |
| 7 | Search #7 | #5 AND #6 [(#1 AND #2) AND (#3 OR #4)] | **1,295** |

### #5 Database: ProQuest

| # | **Filters** | **Filters: Full-text, English, Bahasa Indonesia, year 2001-current, NOT: review**, **book, report, single case, documents, opinion.** | **n** |
| --- | --- | --- | --- |
| 1 | Concept 1: erectile dysfunction | (‘Erectile Dysfunction’ OR ‘erectile disfunction’ OR ‘impotence’ OR ‘impotent’ OR ‘male impotence’ OR ‘male impotent’ OR ‘male sexual impotence’ OR ‘male sexual impotent’ OR ‘male sexual dysfunction’ OR ‘male sexual disfunction’) AND mesh.Exact("Erectile Dysfunction" OR "Impotence" OR "Male")  Additional limits - Date: From 2001 to 2021; Document type: Article; Language: English | 1,136 |
| 2 | Concept 2: diabetes mellitus | (‘diabetes mellitus’ OR ‘type-2 diabetes mellitus’ OR ‘diabetes mellitus type-2’ OR ‘type-2 diabetes’ OR DM OR ‘hyperglycaemia’ OR ‘adult onset diabetes’) AND mesh.Exact("Diabetes Complications" OR "Diabetes Mellitus")  Additional limits - Date: From 2001 to 2021; Document type: Article; Language: English | 3,119 |
| 3 | Concept 3: screening | ‘screen’ OR ‘screening’ OR ‘discuss’ OR ‘discussion’ OR ‘diagnose’ OR ‘diagnosis’ OR ‘management’ OR ‘manage’  Additional limits - Date: From 2001 to 2021 | 532,063 |
| 4 | Concept 4: barriers | experienc** OR barrier OR barriers OR challeng** OR facilitat** OR determinan OR determinants OR treatment OR intervention  Additional limits - Date: From 2001 to 2021 | 566,354 |
| 5 | Search #5 | #1 AND #2 | 33 |
| 6 | Search #6 | #3 OR #4 | 653,508 |
| 7 | Search #7 | #5 AND #6 [(#1 AND #2) AND (#3 OR #4)] | **33** |

### #6 Database: PsychInfo via Ovid

| # | **Filters** | **Filters: Full-text, English, Bahasa Indonesia, year 2001-current, NOT: review**, **book, report, single case, documents, opinion.** | **n** |
| --- | --- | --- | --- |
| 1 | Concept 1: erectile dysfunction | (Erectile Dysfunction or erectile disfunction or impotence or impotent or male impotence or male impotent or male sexual impotence or male sexual impotent or male sexual dysfunction or male sexual disfunction).mp. [mp=title, abstract, heading word, table of contents, key concepts, original title, tests & measures, mesh]  all journals and human and yr="2001 - 2021" | 3022 |
| 2 | Concept 2: diabetes mellitus | (diabetes mellitus or type-2 diabetes mellitus or diabetes mellitus type-2 or type-2 diabetes or DM or hyperglycaemia or adult onset diabetes).mp. [mp=title, abstract, heading word, table of contents, key concepts, original title, tests & measures, mesh]  all journals and human and yr="2001 - 2021" | 14,144 |
| 3 | Concept 3: screening | (screen or screening or discuss or discussion or diagnose or diagnosis or management or manage).mp. [mp=title, abstract, heading word, table of contents, key concepts, original title, tests & measures, mesh]  all journals and human and yr="2001 - 2021" | 473,739 |
| 4 | Concept 4: barriers | (experienc* or barrier or barriers or challeng* or facilitat* or determinan or determinants or treatment or intervention).mp. [mp=title, abstract, heading word, table of contents, key concepts, original title, tests & measures, mesh]  all journals and human and yr="2001 - 2021" | 954,441 |
| 5 | Search #5 | #1 AND #2 | 154 |
| 6 | Search #6 | #3 OR #4 | 1,190,888 |
| 7 | Search #7 | #5 AND #6 [(#1 AND #2) AND (#3 OR #4)] | **90** |

### #7 Database: MedLine via Ovid

| # | **Filters** | **Filters: Full-text, English, Bahasa Indonesia, year 2001-current, NOT: review**, **book, report, single case, documents, opinion.** | **n** |
| --- | --- | --- | --- |
| 1 | Concept 1: erectile dysfunction | (Erectile Dysfunction or erectile disfunction or impotence or impotent or male impotence or male impotent or male sexual impotence or male sexual impotent or male sexual dysfunction or male sexual disfunction).mp. [mp=title, abstract, original title, name of substance word, subject heading word, floating sub-heading word, keyword heading word, organism supplementary concept word, protocol supplementary concept word, rare disease supplementary concept word, unique identifier, synonyms]  limit 1 to (humans and yr="2001 - 2021") | 24,527 |
| 2 | Concept 2: diabetes mellitus | (diabetes mellitus or type-2 diabetes mellitus or diabetes mellitus type-2 or type-2 diabetes or DM or hyperglycaemia or adult onset diabetes).mp. [mp=title, abstract, original title, name of substance word, subject heading word, floating sub-heading word, keyword heading word, organism supplementary concept word, protocol supplementary concept word, rare disease supplementary concept word, unique identifier, synonyms]  limit 1 to (humans and yr="2001 - 2021") | 290,551 |
| 3 | Concept 3: screening | (screen or screening or discuss or discussion or diagnose or diagnosis or management or manage).mp. [mp=title, abstract, original title, name of substance word, subject heading word, floating sub-heading word, keyword heading word, organism supplementary concept word, protocol supplementary concept word, rare disease supplementary concept word, unique identifier, synonyms]  limit 1 to (humans and yr="2001 - 2021") | 3,315,676 |
| 4 | Concept 4: barriers | (experienc* or barrier or barriers or challeng* or facilitat* or determinan or determinants or treatment or intervention).mp. [mp=title, abstract, original title, name of substance word, subject heading word, floating sub-heading word, keyword heading word, organism supplementary concept word, protocol supplementary concept word, rare disease supplementary concept word, unique identifier, synonyms]  limit 1 to (humans and yr="2001 - 2021") | 4,125,945 |
| 5 | Search #5 | #1 AND #2 | 1,256 |
| 6 | Search #6 | #3 OR #4 | 5,978,502 |
| 7 | Search #7 | #5 AND #6 [(#1 AND #2) AND (#3 OR #4)] | **811** |

### #8 Database: Portal Garuda

| # | **Filters** | **Filters: Full-text, English, Bahasa Indonesia, year 2001-current, NOT: review**, **book, report, single case, documents, opinion.** | **n** |
| --- | --- | --- | --- |
| 1 | Concept 1: erectile dysfunction | Disfungsi ereksi OR impoten  Filter: From 2001 to 2021 | 57 |
| 2 | Concept 2: diabetes mellitus | Diabetes  Filter: From 2001 to 2021 | 5,669 |
| 3 | Concept 3: screening | Skrining OR diskus OR diagnosis OR diagnose OR manajemen  Filter: From 2001 to 2021 | 25,403 |
| 4 | Concept 4: barriers | hambatan OR pengalaman OR tantangan OR determinan OR talaksana OR Intervensi  Filter: From 2001 to 2021 | 11,898 |
| 5 | Search #5 | #1 AND #2 | 17 |
| 6 | Search #6 | #3 OR #4 | **0** |
| 7 | Search #7 | #5 AND #6 [(#1 AND #2) AND (#3 OR #4)] | **17** |

### #Recap:

| # | **Filters** | **Filters: Full-text, English, Bahasa Indonesia, year 2001-current, NOT: review**, **book, report, single case, documents, opinion.** | **Included** | **Excluded** |
| --- | --- | --- | --- | --- |
| 1 | Total Findings | Studies imported for screening | 3,468 | 0 |
| 2 | Duplication Removal | Duplication Removal using Covidence | 2,528 | 1,361 |
| 3 | Title & Abstract Screening | Excluded via manual screening using Covidence | 425 | 2,103 |
|  |  | In Conflict (FE & SH) | 421 | - |
|  |  | Conflict resolution by discussion (JB) | 421 | 374 |
| 4 | Eligibility Screening | SH & JB | 47 | 41 |
| 5 | Included | Full-text for assessment & analysis | 6 | 0 |
| 6 | Manual reference search | Finding potential addition | 12 | 12 |
| 7 | Final inclusion |  | 6 | 0 |
