# Supplementary material for: The experiences and barriers in addressing type 2 diabetes mellitus-associated erectile dysfunction: a mixed method systematic review
Source: Syst Rev. 2023 Aug 10;12:138. doi: 10.1186/s13643-023-02303-4 (PMC10416416; doi:10.1186/s13643-023-02303-4)
Supplement: Supplementary file 4 — Additional file 4. Eligibility assessment. [file 13643_2023_2303_MOESM4_ESM.docx]

**Supplementary file 3** – 47 records undertaking full-text review

| No. | Title | Authors | #1 - Full-text retrieval | #2 - Exclusion due to language and type of paper | #3 – Full-text assessment |
| --- | --- | --- | --- | --- | --- |
| 1 | The willingness of Saudi men with type 2 diabetes to discuss erectile dysfunction with their physicians and the factors that influence this. | Almigbal, Turky H.; Schattner, Peter | Y |  | Y |
| 2 | Powerlessness, social support, and glycemic control in Korean adults with type 2 diabetes. | An, Gyeong-Ju; Kim, Mi-Ja | Y |  | Y |
| 3 | Erectile dysfunction and diabetes in Conakry (Guinea): frequency and clinical characteristics from 187 diabetic patients. | Balde, N. M.; Diallo, A. B.; Balde, M. C.; Kake, A.; Diallo, M. M.; Diallo, M. B.; Maugendre, D. | Y | French Language | N |
| 4 | Diabetes and male sexual health: an unmet challenge. | Barnard‚ÄêKelly, Katharine; Meeking, Darryl; Cummings, Michael; Reidy, Claire; Scibilia, Renza; Aldred, Chris; Naranjo, Diana | Y |  | Y |
| 5 | New treatment options for erectile dysfunction in patients with diabetes mellitus. | Basu, A.; Ryder, R. E. | Y | Book section | N |
| 6 | Erectile dysfunction in patients with diabetes: Patients' perspective on barriers experienced and views on how to approach this subject. | Beeharry, D.; Ormerod, H.; Farag, J.; Weston, P.; Sharma, D. | Y | Conference Abstract | N |
| 7 | Erectile dysfunction in diabetes: providing a nurse-led referral service. | Boyd, M. | Y | Audit Report | N |
| 8 | The issue hiding under the sheets. | Cameron, T. |  | Comments/Opinion | N |
| 9 | Erectile dysfunction in diabetes mellitus: Its magnitude and predictors, integration in a bio-psycho-social model. | Chakraborty, K.; Mandal, M.; Chatterjee, S.; Makhal, M.; Basak, G. C. | Full-text not found |  | Y |
| 10 | "My wife's mistrust. That's the saddest part of being a diabetic": A qualitative study of sexual well-being in men with Type 2 diabetes in sub-Saharan Africa. | Cooper, Sara; Leon, Natalie; Namadingo, Hazel; Bobrow, Kirsten; Farmer, Andrew J. | Y |  | Y |
| 11 | The role of patient education in the prevention and management of type 2 diabetes: an overview. | Coppola, Adriana; Sasso, Loredana; Bagnasco, Annamaria; Giustina, Andrea; Gazzaruso, Carmine | Y | Journal Overview | N |
| 12 | Optimizing treatment for diabetes mellitus induced erectile dysfunction. | Costabile, R. A. | Y | Expert Opinion/Literature Review? | N |
| 13 | The role of the general practitioner in men's health | David, Janine | Y | Expert Opinion | N |
| 14 | Erectile dysfunction and quality of life in type 2 diabetic patients: a serious problem too often overlooked. | De Berardis, G.; Franciosi, M.; Belfiglio, M.; Di Nardo, B.; Greenfield, S.; Kaplan, S. H.; Pellegrini, F.; Sacco, M.; Tognoni, G.; Valentini, M.; Nicolucci, A. | Y |  | Y |
| 15 | Identifying patients with type 2 diabetes with a higher likelihood of erectile dysfunction: The role of the interaction between clinical and psychological factors. | De Berardis, G.; Pellegrini, F.; Franciosi, M.; Belfiglio, M.; Di Nardo, B.; Greenfield, S.; Kaplan, S. H.; Rossi, M. C. E.; Sacco, M.; Tognoni, G.; Valentini, M.; Nicolucci, A. | Y |  | Y |
| 16 | Clinical and psychological predictors of incidence of self-reported erectile dysfunction in patients with type 2 diabetes. | De Berardis, G.; Pellegrini, F.; Franciosi, M.; Belfiglio, M.; Di Nardo, B.; Greenfield, S.; Kaplan, S. H.; Rossi, M. C.; Sacco, M.; Tognoni, G.; Valentini, M.; Nicolucci, A. | Y |  | Y |
| 17 | Evaluation and treatment of erectile dysfunction in men with diabetes mellitus. | Dey, J.; Shepherd, M. D. | Y | Expert Opinion/Supplement | N |
| 18 | The multinational Men's Attitudes to Life Events and Sexuality study: the influence of diabetes on self-reported erectile function, attitudes and treatment-seeking patterns in men with erectile dysfunction. | Eardley, I.; Fisher, W.; Rosen, R. C.; Niederberger, C.; Nadel, A.; Sand, M. | Y |  | Y |
| 19 | Management of erectile dysfunction in diabetic subjects: results from a survey of 400 diabetes centres in Italy. | Fedele, D.; Coscelli, C.; Cucinotta, D.; Forti, G.; Santeusanio, F.; Fiori, G.; Velon√†, T.; Lavezzari, M. | Full-text not found |  | N |
| 20 | Real-world observational results from a database of 48 million men in the United States: Relationship of cardiovascular disease, diabetes mellitus and depression with age and erectile dysfunction. | Goldstein, Irwin; Chambers, Richard; Tang, WingYu; Stecher, Vera; Hassan, Tarek | Y |  | Y |
| 21 | How often do we ask about erectile dysfunction in the diabetes review clinic? Development of a neuropathy screening tool. | Grant, P. S.; Lipscomb, D. | Y |  | Y |
| 22 | How often do we ask about erectile dysfunction in the diabetes review clinic? | Grant, Paul S.; Lipscomb, David | Y | Duplication | N |
| 23 | The Experience of Indonesian Men Living with Type-2 Diabetes Mellitus and Erectile Dysfunction: A Semi-structured Interview Study. | Hadisuyatmana, Setho; Efendi, Ferry; Has, Eka Mishbahatul Marah; Wahyuni, Sylvia Dwi; Bauer, Michael; Boyd, James H.; Reisenhofer, Sonia | Y |  | Y |
| 24 | Screening for erectile dysfunction as part of periodic examination programs - concept and implementation. | Heruti, R. J.; Yossef, M.; Shochat, T. | Y |  | Y |
| 25 | Windows of opportunity: a holistic approach to men's health. | Holden, C. A.; Allan, C. A.; McLachlan, R. I. | Y | Expert Opinion | N |
| 26 | Evaluation of a progressive treatment program for erectile dysfunction in patients with diabetes mellitus. | Israilov, S.; Shmuely, J.; Niv, E.; Engelstein, D.; Livne, P.; Boniel, J. | Y |  | Y |
| 27 | Erectile dysfunction, like diabetes, should be considered a 'cardiovascular equivalent'. | Jackson, G. | Y | Editorial/Comments | N |
| 28 | Patterns and Their Correlates of Seeking Treatment for Erectile Dysfunction in Type 2 Diabetic Patients. | Jiann, Bang-Ping; Lu, Chih-Chen; Lam, Hing-Chung; Chu, Chih-Hsun; Sun, Chun-Chin; Lee, Jenn-Kuen | Y |  | Y |
| 29 | A Comprehensive Review of Erectile Dysfunction in Men with Diabetes. | Kamenov, Z. A. | Y | Expert Opinion | N |
| 30 | Assessment of cardiovascular risk in patients with erectile dysfunction: focus on the diabetic patient. | Kloner, R. A. | Y | Expert Opinion | N |
| 31 | Prevalence, correlates, attitude and treatment seeking of erectile dysfunction among type 2 diabetic Chinese men attending primary care outpatient clinics. | Lo, W. H.; Fu, S. N.; Wong, C. K.; Chen, E. S. | Y |  | Y |
| 32 | What do men with diabetes and erectile dysfunction think about the services they receive? | McMurray, M.; Davies, M. | Y |  | Y |
| 33 | Sexual morbidity in men with diabetes. | Morrison, C. L.; Morrison, G.; Purewal, T. S.; Weston, P. J. | Y | Conference Abstract | N |
| 34 | The experiences of South Asian men with diabetes and erectile dysfunction (ED). | O'Brien, C.; Frizelle, D.; Gardner, P.; Farrell, K. | Y | Conference Abstract | N |
| 35 | Erectile dysfunction: they don't talk, we don't ask. | Rakovac Tisdall, A.; King, T. F. J.; Mahmood, W. A. W.; Keat, C. S.; Ali, R.; Abdin, R.; Koo, C. M.; Alali, M.; Sreenan, S.; McDermott, J. H. | Y | Letter to the editor | N |
| 36 | How much of a priority is treating erectile dysfunction? A study of patients' perceptions. | Rance, J.; Phillips, C.; Davies, S.; O'Malley, B.; Zaman, Q.; Price, D. | Y |  | Y |
| 37 | How much of a priority is treating erectile dysfunction? A study of patients' perceptions. | Rance, J.; Phillips, C.; Davies, S.; O'Malley, B.; Zaman, Q.; Price, D. | Y | Duplication | N |
| 38 | Say yes to intimacy. Treatment options for erectile dysfunction. | Rice, D. | Full-text not found |  | N |
| 39 | Men's Health, Low Testosterone, and Diabetes Individualized Treatment and a Multidisciplinary Approach. | Rice, Donna; Brannigan, Robert E.; Campbell, R. Keith; Fine, Shari; Jack, Leonard, Jr.; Nelson, Joseph B.; Regan-Klich, Janet | Y | Article Supplement | N |
| 40 | Effectiveness of a PLISSIT model intervention in patients with type 2 diabetes mellitus in primary care: design of a cluster-randomised controlled trial. | Rutte, A.; van Oppen, P.; Nijpels, G.; Snoek, F. J.; Enzlin, P.; Leusink, P.; Elders, P. J. | Y |  | Y |
| 41 | Type 2 Diabetes Patients' Needs and Preferences for Care Concerning Sexual Problems: A Cross-Sectional Survey and Qualitative Interviews. | Rutte, A.; Welschen, L. M.; van Splunter, M. M.; Schalkwijk, A. A.; de Vries, L.; Snoek, F. J.; Enzlin, P.; Nijpels, G.; Elders, P. J. | Y |  | Y |
| 42 | Symptom report and treatment experience of hypogonadal men with and without type 2 diabetes in a United States health plan. | Shortridge, E. F.; Polzer, P.; Donga, P.; Wade, R. L. | Y |  | Y |
| 43 | Hypogonadism, Erectile Dysfunction, and Type 2 Diabetes Mellitus: What the Clinician Needs to Know. | Tamler, Ronald; Deveney, Tatiana | Y | Review | N |
| 44 | The pharmacist's role in improving the treatment of erectile dysfunction and its underlying causes. | Taylor, D. G.; Giuliano, F.; Hackett, G.; Hermes-DeSantis, E.; Kirby, M. G.; Kloner, R. A.; Maguire, T.; Stecher, V.; Goggin, P. | Y | Expert Opinion | N |
| 45 | My Thing Is Dead: Experience of Dealing with Diabetic Erectile Dysfunction of Northern Thai Men. | Thongtaeng, Pulawit; Fongkaew, Warunee; Sansiriphun, Nantaporn; Chaloumsuk, Nonglak | Y |  | Y |
| 46 | Diabetes-induced erectile dysfunction: epidemiology, pathophysiology and management. | Thorve, Vrushali S.; Kshirsagar, Ajay D.; Vyawahare, Neeraj S.; Joshi, Vipin S.; Ingale, Kundan G.; Mohite, Reshma J. | Y | Expert Opinion | N |
| 47 | Management of erectile dysfunction in diabetic patients. | Ziegler, D. | Full-text not found |  | N |
