# Supplementary material for: The experiences and barriers in addressing type 2 diabetes mellitus-associated erectile dysfunction: a mixed method systematic review
Source: Syst Rev. 2023 Aug 10;12:138. doi: 10.1186/s13643-023-02303-4 (PMC10416416; doi:10.1186/s13643-023-02303-4)
Supplement: Supplementary file 5 — Additional file 5. JBI's data extraction forms. [file 13643_2023_2303_MOESM5_ESM.pdf]

Additional file 5:
JBI Mixed Methods Data Extraction Form following a Convergent Integrated Approach

Reviewer: Setho Hs

Date: Tue, 14 June 2022

Author(s) of the Publication: Jiann, Bang-Ping; Lu, Chih-Chen; Lam, Hing-Chung; Chu, Chih-Hsun; Sun, Chun-Chin; Lee, Jenn-Kuen

Year: 2009

Journal: Journal of Sexual Medicine

Record Number: #1450

Type of study

- [x] Quantitative study
- ○ Qualitative study
- ○ Mixed methods study

Methodology: (e.g. randomized controlled trial, phenomenology) Cross-sectional - Survey

Number of participants: 916

Characteristics of participants

916 Taiwan-Chinese men aged 26 to 85 years old who lived with T2DM and visited the outpatient department (OPD) of endocrinology under the diagnosis of diabetes mellitus (DM) at Kaoshiung Veterans General Hospital during January 2004 to May 2006.

Phenomena of interest

Investigating the spare research issue around prevalence of ED in T2DM men and to look whether theythe men were bothered by it or not are. Thus, the study was conducted to evaluate the prevalence of ED in type 2 diabetic patients, their treatment-seeking patterns, and factors affecting them.

Setting and other context-related information (e.g. cultural, geographical)

One Chinese-Taiwan Outpatient Department at a General Hospital in Taiwan was used to source the participating men.

Outcomes or findings of significance to the review objectives

For a quantitative study

**Results**	According to SHIM score, the majority (83.9%) of the subjects had severely suffered from ED (43.6%) severe. Those with ED were older in age (67.5 + 12.5 years) and were more likely to have comorbidities, including hypertension (49.9%), coronary artery disease (14.1%), and depression (4.6%). Their ED prevalence and severity were positively correlated with increasing age (P <0.05). Of the subjects with ED, mostly reported to be bothered by it (65.1%), either it is modestly (13.1%) or highly bothered (14.6%). Of the subjects with ED, the majority of them had interests in ED treatment (50.5%), either its some (38.3%) and much interest (12.2%). In groups of having more severe ED than mild, those younger than 66 years had a higher interest in ED treatment than those older than 65 years (P <0.05). However, only 27.8% (194/699) of the subjects with ED had ever sought treatment for ED through: counselling with a Western physician (14.2%); taking medication from over the counter (11.6%); adopting diet control or taking exercise (7.0%); or counselling with a traditional Chinese medicine practitioner (5.3%).<br>Of 701 subjects with ED, the majority of them (56.6%) wished to discuss ED with their doctors. Although, only few (16.7%) of them had ever discussed ED problem with their doctors due to feeling embarrassed to talk about it (42.8%), perceiving the treatments were no use and not effective (23.2%), the treatment will harm their general health (7.7%), burdened by the financial constraints (7.0%), perceiving sex is not important (4.8%), rejecting to admit their erectile problem (4.1%), and feeling too old to ask for a help (3.0%). Despite, few (7.9%) of them had been asked about this subject by their doctors and most participants with ED (90.4%) and at the same time wishing to discuss it with their DM doctors.

For a qualitative study

**Themes or Subtheme**	**Illustration (a direct quotation from a participant, an observation or other supporting data from the paper)**

Author's conclusion

The prevalence of ED in diabetic patients was high and most of them felt bothered and had inter- est in treatment of it, but only few of them had actually ever sought professional help. ED severity was the major determinant of whether they felt bothered or sought treatment for it. Embarrass- ment related to discussing ED and misinformation about ED treatment were the main causes for not seeking professional help, and most patients wanted their doctors to initiate discussion of ED. Doctors taking care of diabetic patients wererecommended to routinely screen for erectile problems.

Reviewer's Comments

This paper identified barriers of ED screening using the men as patient's point of view. Expectations of having a discussion was expressed, despite only few doctors addressed ED.

Additional file 3:
JBI Mixed Methods Data Extraction Form following a Convergent Integrated Approach

Reviewer: Setho Hs

Date: Tue, 14 June 2022

Author(s) of the Publication: Lo, W. H.; Fu, S. N.; Wong, C. K.; Chen, E. S.

Year: 2014

Journal: Asian Journal of Andrology

Record Number: #2503

Type of study

- ☒ Quantitative study
- ○ Qualitative study
- ○ Mixed methods study

Methodology: (e.g. randomized controlled trial, phenomenology) Cross-sectional - Survey

Number of participants: 603

Characteristics of participants

Chinese-Hongkong men aged 60.5 +/- 10.5 years who received care from 10 public outpatient clinics between March to May 2012 were participating in the study.

Phenomena of interest

Considering the important yet the difficulty in diagnosing ED in men with T2DM, the study was conducted to find out the prevalence of ED by the use of structural questionnaires, the reliability of the questionnaires as indication of prevalence, and to find out the factors that correlated with T2DM patients' attitude toward ED and their expectation for ED management in the primary care setting.

Setting and other context-related information (e.g. cultural, geographical)

Chinese men who came for a service at 10 Hong Kong's government general outpatient clinics were invited to fill-in a self-administered questionnaire while waiting for service in a waiting hall.

Outcomes or findings of significance to the review objectives

For a quantitative study

**Results**	Although most participating men (79.1%) were found to have ED, only half of them were aware that they have ED (54.7%). Amongst those who have ED, many of them (45.4%) perceived the sexual problem as a natural consequence of ageing, and one in every three respondents (29.5%) viewed ED as an illness that requires treatment or as a secondary consequence of a disease (12.6%). <10% of the subjects with ED had ever sought help from any doctor regardless of degree of severity. However, 76.1% of the subjects preferred receiving management from doctors should they be diagnosed with ED. For management options, 41.7% would like assessment by doctor, followed by management of potential underlying cause (37.8%), referral to specialist (27.5%), education and information on ED (23.9%), prescription of phosphodiesterase type 5 inhibitors (PDE-5) (16.9%) and referral to counselling service (6.7%).

For a qualitative study

**Themes or Subtheme**	**Illustration (a direct quotation from a participant, an observation or other supporting data from the paper)**

Author's conclusion

T2DM men with ED wanted management from doctors, but they seldom seek help actively. Most of them expected doctors to offer assessment and management of potential underlying causes, while desire for counseling and education services and drug therapy was low.

Reviewer's Comments

This study was conducted partly to look for factors that associated with T2DM patients atituede toward help seeking behaviour. The participants of this study perceived that ED was ageing consequences, thus reasoned the low help seeking behaviour. Further, the article explored participant's expectations that recommends future service.

Additional file 3:
JBI Mixed Methods Data Extraction Form following a Convergent Integrated Approach

Reviewer: Setho Hs

Date: Tue, 14 June 2022

Author(s) of the Publication: Shortridge, E. F.; Polzer, P.; Donga, P.; Wade, R. L.

Year: 2015

Journal: International Journal of Clinical Practice

Record Number: #2805

Type of study

- ☒ Quantitative study
- ○ Qualitative study
- ○ Mixed methods study

Methodology: (e.g. randomized controlled trial, phenomenology) Cross-sectional - Survey

Number of participants: 19 (w/ HG+DM)

Characteristics of participants

19 out of 93 participants were hypergonadism and type-2 diabetics, that were involved form the Reliant Medical Group in central Massachusetts, spread across 20 sites in the USA. The participants' aged 53.94 (+/-10.83) years, mostly Caucasians (15 of 19), college educated or higher (15 of 19).

Phenomena of interest

To examine symptom reporting and patterns of TRT use and men's perceptions of symptoms and treatment, and further understand of the HG patients' perspectives, contrasting those with and without T2DM.

Setting and other context-related information (e.g. cultural, geographical)

The data for analysis were gathered from men who were customer of the Reliant Group that was situated in 20 sites accross the USA.

Outcomes or findings of significance to the review objectives

For a quantitative study

**Results**
Of the 19 men with HG and T2DM, ED was found in 17 men as bothersome symptom, while decreased sex drive (11) and feeling tired (12) add as the reasons for the men to seek help. Most of the men did not receive TRT due to receiving drug therapy (17). Despite, all of them at least had one TRT in the past. Although they prefered to continue the therapy, most of them (13) discontinued the treatment due to lack efficacy (5), high cost of treatment (4), and inconvenient (3).

For a qualitative study

**Themes or Subtheme**	**Illustration (a direct quotation from a participant, an observation or other supporting data from the paper)**

Author's conclusion

ED was the primary reason for all men seeking care. Men with HG only were less likely than those with both HG and T2DM to report that they were currently experiencing key symptoms compared with when they were first diagnosed, regardless of TRT utilisation, and were less likely to report ED as a current symptom.

Reviewer's Comments

This study identified some of the barriers experienced by the men who were seeking help from ED. Amongst the most reported, cost of treatment, lack of TRT efficacy, and inconvenient treatment were the main barriers that contributed to a deliberate decision for the men to discontinue treatment.

Additional file 3:
JBI Mixed Methods Data Extraction Form following a Convergent Integrated Approach

Reviewer: Setho Hs
Date: Mon, 13 June 2022
Author(s) of the Publication: Turky H. Almigbal, Peter Schattner
Year: 2018
Journal: Plos One
Record Number: #3226

Type of study

- ⊗ Quantitative study
- ○ Qualitative study
- ○ Mixed methods study

Methodology: (e.g. randomized controlled trial, phenomenology) Cross-sectional - Survey

Number of participants: 309 Saudi Arabian Men with T2DM

Characteristics of participants

309 men with tehe median age of 60 years and the median of duration of living with diabetes was 10 years

Phenomena of interest

(1) To find out the proportion of Saudi men with type 2 diabetes who have been asked about ED in the last year by their physicians in hospital-based primary care clinics (2) To determine the willingness of Saudi men with type-2 diabetes to discuss ED with their physicians and the factors that influence their willingness to discuss this issue.

Setting and other context-related information (e.g. cultural, geographical)

Hospital-based primary care clinics of a university hospital, Kingdom of Saudi Arabia

Outcomes or findings of significance to the review objectives

For a quantitative study

**Results**
Only few men had been questioned by their physicians about ED (9.7%), despite the most of them (84.8%) were expecting to discuss the issue. The participants who were unwilling to discuss ED were those complaining of severe ED or those aged older than 60. "Embarasing the doctor", "ED is a personal issue", "too old for now", "feeling embarrassed to talk about it", "too sick now to address ED issues", "no effective treatment is available", and "my doctor is too young to discuss my ED" are identified as the perceived barriers for the men to manage T2DMED with the doctor. After adjusting the age and severity of ED, it is predicted that "it may embarrass my doctor" and "it is a personal issue" as the men's barriers. However, most participants who had never been asked about ED were willing to discuss it with the doctor.

For a qualitative study

**Themes or Subtheme**	**Illustration (a direct quotation from a participant, an observation or other supporting data from the paper)**

Author’s conclusion

ED is a highly prevalent condition among patients who have type 2 diabetes. Most of these patients are not asked about ED within the last year of attending a clinic, even though most are willing to discuss it with their physicians. Many patients’ barriers to discussing ED have been identified, including being older and suffering from more severe ED, with these patients being less willing to discuss this with their physicians. Further research is needed to explore the bar- riers which prevent physicians from discussing ED with their patients who have diabetes.

Reviewer’s Comments

Age and severity of ED are identified as a predictor for men's willingness to discuss about ED with their doctor. Patients who were and feeling old, and having a severe ED were less likely to discuss about ED as compared with those who were younger and have milder ED, despite the majority of the old patients still interested in sexual activity.<br>Despite possibilities of confounding factors may have had an impact on the analysis, the use of strict questions in the questionnaire may limit patient's non-focused thoughts. However, that the questionnaires used were piloted and validated, this improve the reliability of the study findings.

Additional file 3:
JBI Mixed Methods Data Extraction Form following a Convergent Integrated Approach

Reviewer: Setho Hs

Date: Tue , 14 June 2022

Author(s) of the Publication:
Cooper, Sara; Leon, Natalie; Namadingo, Hazel; Bobrow, Kirsten; Farmer, Andrew J.

Year: 2018

Journal:
Plos One

Record Number: #3256

Type of study

- o Quantitative study
- X Qualitative study
- o Mixed methods study

Methodology: (e.g. randomized controlled trial, phenomenology) Descriptive Qualitative

Number of participants: 47

Characteristics of participants

47 participants were included in the study: 11 from Cape Town, 15 from Johannes- burg and 21 from Lilongwe. Mean age of all participants was 55.3 years (SD: 11.2). The youngest participant was 28 years old; the oldest 78 years. All FGDs comprised a mix of ages: ages ranged from 28–69 years in the Johannesburg FGD; 45– 65 years in the Cape Town FGD; and 32–70 years in one FGD in Lilongwe and 41–68 years in the other Lilongwe FGD. Nearly half (48.9%) of participants had been living with diabetes for between 1 and 5 years and hypertension was t he most common co-morbid condition. Most participants (80.9%) were taking oral medication only for their diabetes

Phenomena of interest

This study explores how men with type 2 diabetes in three sub-Saharan African settings (Cape Town and Johannesburg, South Africa; Lilongwe, Malawi) perceived and experienced sexual functioning and sexual well-being, and the biopsychosocial contexts in which these occur and are shaped.

Setting and other context-related information (e.g. cultural, geographical)

The study was conducted in three major cities of two sub-saharan countries of Africa,<br>Low and Middle Income Countries.

Outcomes or findings of significance to the review objectives

For a quantitative study

**Results**

For a qualitative study

**Themes or Subtheme**	**Illustration (a direct quotation from a participant, an observation or other supporting data from the paper)**
	Detailed in the table as a separate file

Author's conclusion

Sexual difficulties emerged as a key and pressing concern for men with diabetes in this study. These difficulties went beyond physiological impediment to intercourse, comprising complex psychological and relational meanings and effects. There is a need for greater awareness on the part of patients, their partners, and healthcare workers of the experiences of sexual difficulties in the context of living with diabetes, and increased efforts to address this constructively. More in-depth research, amongst both men and women, is needed to better understand the impor- tance of sexual functioning and sexual well-being in supporting diabetes self-management, especially in light of the rising prevalence of diabetes in LMIC settings.

Reviewer's Comments

This study explored the issues around the lived experience of sexual difficulties, associated impact, meanings, values and priorities amongst men with diabetes; with the aim to inform strategises to better respond to the sexual concerns and support needs of people with diabetes. Not feeling supported by the HCP was discussed using men point of view. The men informed of not having supportive information, advice, and support they needed to cope with ED. Most men had not raised this issue to the HCP and claimed unsatisfied with the responses they recived. They reported the absence of discussion, information around T2DMED. Feeling 'awkward' and 'uncomfortable' were raised as the reasons that voided the discussion. Although the men also recognized that the HCP were lack capacity, overburdened and having limited time for consultation. Among the South African participants, reported the negative past experience (i.e., being scolded, shamed, and blamed) have caused fear of speaking their situations to the HCP; thus, remained silence. For some who had raised the topic reported that they had received unhelpful and hurtful responses that were dismissive and punitive, such as shaming, and blaming.

Additional file 3:
JBI Mixed Methods Data Extraction Form following a Convergent Integrated Approach

Reviewer: Setho Hs
Date: Thu, 16 June 2022
Author(s) of the Publication:
Hadisuyatmana, Setho; Efendi, Ferry; Has, Eka Mishbahatul Marah; Wahyuni, Sylvia Dwi; Bauer, Michael; Boyd, James H.; Reisenhofer, Sonia
Year: 2021
Journal:
Sexuality & Disability
Record Number: #3809

Type of study

- ○ Quantitative study
- ☒ Qualitative study
- ○ Mixed methods study

Methodology: (e.g. randomized controlled trial, phenomenology) Descriptive Qualitative

Number of participants: 12

Characteristics of participants

Indonesian men aged between 45 and 53 years old living with T2DM ranged from 1 to 13 years. Most were married, only one man, a widower, was a single. All participants were employed or self-employed or lived by opening a small shop.

Phenomena of interest

That little was known about the support available for Indonesian men with T2DM who are at risk of ED, and what health services and support they expect to receive, the study attempted to understand how sexual relationships are experienced and lived by men with T2DM in Indonesia, their experiences of ED and associated healthcare.

Setting and other context-related information (e.g. cultural, geographical)

The participants were Indonesian men who routinely accessed public health centre in the second biggest city of Indonesia.

Outcomes or findings of significance to the review objectives

For a quantitative study

**Results**

For a qualitative study

**Themes or Subtheme**	**Illustration (a direct quotation from a participant, an observation or other supporting data from the paper)**
	Detailed in the table as a separate file

Author's conclusion

Reviewer's Comments

